# Features of Solution Behavior of Polymer Stars with Arms of Poly-2-alkyl-2-oxazolines Copolymers Grafted to the Upper Rim of Calix[8]arene

**DOI:** 10.3390/polym13152507

**Published:** 2021-07-29

**Authors:** Tatyana Kirila, Alina Amirova, Alexey Blokhin, Andrey Tenkovtsev, Alexander Filippov

**Affiliations:** Institute of Macromolecular Compounds of the Russian Academy of Sciences, Bolshoy Pr. 31, 199004 Saint Petersburg, Russia; aliram.new@gmail.com (A.A.); 44stuff44@gmail.com (A.B.); avt@hq.macro.ru (A.T.); afil@imc.macro.ru (A.F.)

**Keywords:** synthesis, star-shaped macromolecules, calix[n]arene, block and gradient copolymers of poly-2-alkyl-2-oxazolines, conformation, thermoresponsibility, self-organization, phase separation

## Abstract

Star-shaped polymers with arms of block and gradient copolymers of 2-ethyl- and 2-isopropyl-2-oxazolines grafted to the upper rim of calix[8]arene were synthesized by the “grafting from” method. The ratio of 2-ethyl- and 2-isopropyl-2-oxazoline units was 1:1. Molar masses and hydrodynamic characteristics were measured using molecular hydrodynamics and optics methods in 2-nitropropane. The arms of the synthesized stars were short and the star-shaped macromolecules were characterized by compact dimensions and heightened intramolecular density. The influence of the arm structure on the conformation of star molecules was not observed. At low temperatures, the aqueous solutions of the studied stars were not molecular dispersed but individual molecules prevailed. One phase transition was detected for all solutions. The phase separation temperatures decreased with a growth of the content of more hydrophobic 2-isopropyl-2-oxazoline units. It was shown that the way of arms grafting to the calix[8]arene core affects the behavior of aqueous solutions of star-shaped poly-2-alkyl-2-oxazoline copolymers. In the case of upper rim functionalization, the shape of calix[8]arene resembles a plate. Accordingly, the core is less shielded from the solvent and the phase separation temperatures are lower than those for star-shaped poly-2-alkyl-2-oxazolines with lower rim functionalization of the calix[8]arene.

## 1. Introduction

New synthetic routes make it possible to obtain well-defined polymers with complex architecture [1,2,3,4] including multiarm stars [5,6,7,8,9,10]. Their behavior in solutions is determined by the chemical structure of the core and arms and the number and length of the latter. The use of copolymers as arms is a convenient way to control the properties of stimulus-sensitive star polymers. In the case of block copolymer arms, the sequence of block attachment affects both the phase separation temperatures and the dimensions of supramolecular structures present in solutions [11,12,13,14]. For example, the variation in solution behavior was observed for triblock copolymer stars with arms consisting of hydrophobic, hydrophilic, and thermosensitive blocks. When the thermosensitive block was located near a core, intramolecular aggregation took place and aggregates with a smaller diameter were formed in comparison with the supramolecular structures formed in solutions of macromolecules with a thermosensitive block in the outer layer [12]. From general considerations, it is clear that the stimulus-sensitivity of copolymer stars depends on the ratio of the components [11,15,16,17,18,19].

Thermoresponsive poly-2-alkyl-2-oxazolines (PAlOx) have been actively studied in recent decades due to the wide potential of their application [20,21,22,23]. One of the ways for using this class of polymers is medicine due to their biocompatibility and non-toxicity, stability in enzyme media [24], and lower critical solution temperature (LCST) near to the human body one. These are the reasons to enhance the investigation of star-shaped PAlOx properties depending on the molecule structure. The optimal conditions have been established, which make it possible to obtain PAlOx stars with a given number and length of arms and, accordingly, to regulate their conformational characteristics and behavior in water–salt solutions, including thermosensitivity and association with low molecular weight compounds. The study star-shaped block PAlOx copolymers revealed that a different sequence of block attachment to the core does not influence the phase separation temperatures, however, it determines the set and dimensions of scattering objects [13]. In the case of a polymer with a more hydrophilic outer block, aggregation processes prevail, while for a polymer star with a more hydrophobic outer block in a wide temperature range, the dominant process is aggregation [25]. The distribution of 2-alkyl-2-oxazoline units along the arm chains also affects the stimulus-sensitivity of copolymer PAlOx, in particular, the temperature of the onset of phase separation *T*_1_ for solutions of a stars with gradient arms is higher than the *T*_1_ value for block copolymer stars [26].

Concerning the macromolecule core, its structure and size have a great effect on the properties of thermoresponsive star-shaped PAlOx. For example, competition between compaction and aggregation processes was observed upon heating of solutions of PAlOx stars with a massive hydrophobic dendrimer core, while aggregation dominates in solutions of PAlOx with a less hydrophobic calix[n]arene core [27]. The use of calixarene derivatives as the branching center of star-shaped polymers is due to bringing the unique ability of calix[n]arenes to complex formation with low molecular weight organic compounds [28,29,30]. Accordingly, a number of calix[n]arene derivatives with functionalization with low molecular weight fragments have been proposed for use in targeted drug delivery systems [31,32,33,34,35,36]. Calixarenes with polymer arms have also been obtained [25,37]. It is also important to point out that polymer stars with a calix[n]arene core are high macromolecular weight objects, components of which are selectively solvated by water. It provides another mechanism for regulating the characteristics of thermoresponsive supramolecular structures.

Note that most studies describe the results for stars in which polymer arms are grafted to the lower rim of calix[n]arenes. At the same time it is known that different positions of functional groups or polymer arms in calix[n]arene lead to a variation in physicochemical properties and self-organization of calix[n]arene derivatives [38,39]. It was shown that the grafting of the homopolymer PAlOx chains at the upper rim of calix[n]arene reduces the phase separation temperatures as compared to polymer stars with arm grafting to the lower rim [39]. The present work was aimed at the analysis of the influence of the arm structure and the configuration of the calix[8]arene (C8A) core on the molecular conformation, the solution behavior, and the self-organization of star-shaped PAlOx copolymers in aqueous solutions upon heating. To solve this problem, eight-arm polymer stars were synthesized and studied. Their arms were block copolymers of poly-2-ethyl-2-oxazoline (PEtOx) and poly-2-isopropyl-2-oxazoline (PiPrOx). These samples differed in the order in which the blocks were attached to the core. In the C8A-(PiPrOx-b-PEtOx) star, the inner block was PiPrOx, and in the C8A-(PEtOx-b-PiPrOx) copolymer, PEtOx was attached to the core. In addition, the star-shaped copolymer C8A-P(EtOx-grad-iPrOx) was studied, the arms of which were gradient copolymers of 2-ethyl-2-oxazoline (EtOx) and 2-isopropyl-2-oxazoline (iPrOx). In these stars, the content of EtOx units decreased with distance from the C8A core. For comparison, a star-shaped homopolymer C8A-PEtOx with PEtOx chains attached to the upper rim of calix[8]arene was studied. It is important that the synthesized star-shaped samples should have a similar arm length in order to avoid the influence of molar mass on the obtained characteristics.

## 2. Materials and Methods

### 2.1. Materials and Reagents

Dialysis bags “CellaSep”, MWCO = 3000 Da (Orange Scientific, Braine-l′Alleud, Belgium) were used for the purification of polymer samples. Monomers, 2-ethyl- and 2-isopropyl-2-oxazolines (Sigma-Aldrich, St. Louis, MO, USA) were distilled over the calcium hydride. Sulfolane (Sigma-Aldrich, St. Louis, MO, USA) was purified by vacuum distillation. Pyrrolidine (Sigma-Aldrich, St. Louis, MO, USA) was distilled over calcium hydride.

### 2.2. Synthesis of Multicenter Macroiniciater

The octafunctional initiator, 5,11,17,23,29,35,41,47-octakis-(chlorosulfonyl)-49,50,51,52,53,54,55,56-octakis-(methoxycarbonylmethoxy)-calix[8]arene, was synthesized following the described scheme [40].

#### 2.2.1. 49,50,51,52,53,54,55,56-Octa(hydroxy)calix[8]arene

The mixture of 10 g (7.7 × 10^−3^ mol) of tert-butylcalix[8]arene, 5.8 g (0.062 mol) of phenol, 12.3 g (0.092 mol) of aluminum chloride, and 150 mL of toluene was stirred for 1 h at ambient temperature, after which it was poured into 170 mL of 0.2 M hydrochloric acid. The organic layer was separated and the solvent was distilled off. The precipitate was washed with 330 mL of methanol, acidified with a few drops of hydrochloric acid, and filtered out. The product was purified by chloroform extraction in Soxhlet apparatus during 24 h. Yield 5.5 g (84%). ^1^H NMR (400 MHz, DMSO, 20 °C): δ (ppm) 6.0–7.0 (m, 24H), 3.5 (s 16H).

#### 2.2.2. 49,50,51,52,53,54,55,56-Octa(methoxy(carbonylmethoxy)calix[8]arene

The mixture of 29 g (0.21 mol) of dry potassium carbonate, 11.2 g (0.067 mol) of dry potassium iodide, 3.45 g (4.1 × 10^−3^ mol) of calix[8]arene, 18 mL (0.21 mol) of methyl chloroacetate, and 180 mL of absolute acetonitril was heated at 80 °C during 24 h. The reaction mixture was poured into 300 mL of water. The product was extracted with diethyl ether (2 × 100 mL), washed with water, and dried (MgSO_4_). After the evaporation product was recrystallized from methanol. Yield 2.1 g (36%). ^1^H NMR (400 MHz, CDCl_3_, 20 °C): δ (ppm) 6.9 (m, 24H), 4.27 (m, 16H), 4.10 (s, 16H), 3.7 (s, 24H).

#### 2.2.3. 5,11,17,23,29,35,41,47-Octachlorosulfonyl-49,50,51,52,53,54,55,56-octa(methoxy(carbonylmethoxy)calix[8]arene

A solution of 2 g (1.41 × 10^−3^ mol) of octa(methoxy-(carbonylmethoxy)) calix[8]arene in 60 mL of chloroform was cooled to −10 °C and 20 mL (0.3 mol) of chlorosulfonic acid was added drop by drop. Then mixture was heated to 50 °C (at about 20 min) and left at this temperature for 20 min. After cooling to room temperature, the mixture was gradually poured into a mixture of 400 mL of ice water and 300 mL of petroleum ether and left for 30 min. The product was filtered off, washed with water, then with petroleum ether, and dried. The crude product was dissolved in a minimum amount of dichloromethane and reprecipitated into petroleum ether. This procedure was repeated twice. Yield: 1.5 g (49%). M.p 170 °C (with decomposition). ^1^H NMR (400 MHz, CDCl3, 20 °C): δ (ppm) 7.6 (m, 16H), 4.2–4.7 (m, 32H), 3.7 (s, 24H). Elemental analysis: Calc. C 44.37%, H 3.72%, Cl 12.47%, S 11.28%. Found C 44.1%, H 4.0%, S 11.6%, Cl 12.8%.

### 2.3. Eight-Arm Star Poly(2-Ethyl-2-Oxazoline-Block-2-Isopropyl-2-Oxazoline) Copolymer Synthesis

The solution of 0.0931 g (0.0421 mmol) of the octafunctional initiator in 2 g (17 mmol) of sulfolane was prepared under the nitrogen atmosphere. The solution was mixed with 1 g (10.1 mmol) of 2-ethyl-2-oxazoline and sealed in a vial. The mixture was kept at 100 °C for 24 h. After that, 1.14 g (10.1 mmol) of 2-isopropyl-2-oxazoline was injected into the vial, which was sealed again and kept at 100 °C for 48 h. Then 1 mL (12.1 mmol) of pyrrolidine was added into the vial and the solution was stirred at 50 °C for 1 h. The polymer was purified by dialysis in 0.1 M sodium hydroxide aqueous solution followed by pure water, then dried at 20 °C, and finally evaporated from its chloroform solution (1.61 g, 72%). ^1^H NMR (CDCl3, δ, ppm): 3.51 (m, 4H), 3.05–2.65 (m, 1H), 2.58–2.20 (m, 2H), 1.11 (m, 9H).

### 2.4. Eight-Arm Star Poly(2-Isopropyl-2-Oxazoline-Block-2-Ethyl-2-Oxazoline) Copolymer Synthesis

The same techniques of synthesis and purification were applied as previously discussed (1.68 g, 75%). ^1^H NMR (CDCl3, δ, ppm): 3.52 (m, 4H), 3.05–2.60 (m, 1H), 2.58–2.20 (m, 2H), 1.11 (m, 9H).

### 2.5. Eight-Arm Star Poly(2-Ethyl-2-Oxazoline-Grad-2-Isopropyl-2-Oxazoline) Gradient Copolymer Synthesis

The solution of 0.1 g (0.0452 mmol) of the octafunctional initiator in 2.5 g (20 mmol) of sulfolane was prepared under the nitrogen atmosphere. Equimolar amounts (10.8 mmol) of 2-ethyl-2-oxazoline and 2-isopropyl-2-oxazoline were mixed and added to the solution, after that it was sealed in a vial. The mixture was kept at 100 °C for 72 h, then 1 mL (12.1 mmol) of pyrrolidine was added into the vial and the solution was stirred at 50 °C for 1 h. The same technique of purification was used as previously discussed (1.95 g, 82%). ^1^H NMR (CDCl3, δ, ppm): 3.50 (m, 4H), 3.00–2.60 (m, 1H), 2.60–2.20 (m, 2H), 1.10 (m, 9H).

The synthesis and characterization of 8-arm of poly(2-ethyl-2-oxazoline) was described early [39].

### 2.6. Hydrolysis of Star-Shaped Polymers

A solution of 0.1 g of star-shaped poly-2-alkyl-2-oxazoline in 5 mL of 1 M hydrochloric acid was heated in a sealed ampoule at 100 °C during 24 h, after which it was evaporated to dryness. The residue was dissolved in 5 mL of ethyl alcohol, dialyzed against sodium bicarbonate (concentration 0.1 mol/L) using CellaSep dialysis bags with MWCO 3500 Da and freeze-dried. The product was dissolved in 15 mL of propionic anhydride, heated at 50 °C during 30 min, and evaporated under reduced pressure.

### 2.7. Characterization of Prepared Star Samples

UV–visible spectra were obtained using the SF-256 (LOMO-Photonika, Saint-Peterburg, Russia) spectrophotometer for ethanol solutions. The NMR spectra were measured on the Bruker AC400 (400 MHz) (Bruker, Billerica, MA, USA) spectrometer using chloroform solutions. Dialysis was conducted using dialysis sacks (CellaSep, Orange Scientific, Braine-l’Alleud, Belgium); MWCO, 3500 Da. Chromatographic analysis was performed on the Shimadzu LC-20AD chromatograph (Shimadzu Corporation, Nishinokyo Kuwabara, Japan) equipped with the TSKgel G5000HHR column (5 μm, 7.8 mm × 300 mm, TosomBioscience, Tokyo, Japan) and light scattering and UV detectors. The mobile phase was a solution of LiBr (0.1 mol/L) in dimethylformamide at 60 °C. Polyethyleneglycol standards were chosen.

### 2.8. Investigation of Molecular-Dispersed Polymer Solutions

Molar mass and hydrodynamic characteristics of the synthesized polymers were obtained by molecular hydrodynamic and optics methods. Measurements were carried out in 2-nitropropane (dynamic viscosity η_0_ = 0.72 cP, density ρ_0_ = 0.982 g·cm^−3^, and refractive index *n*_0_ = 1.394) at 21 °C.

Dynamic and static light scattering was studied using the Photocor Complex setup (Photocor Instruments Inc., Moscow, Russia); the light source was a Photocor-DL diode laser with a wavelength λ = 658.7 nm. The correlation function of the scattered light intensity was obtained using the Photocor-PC2 correlator with 288 channels and processed using the DynaLS software (ver. 8.2.3, SoftScientific, Tirat Carmel, Israel).

**Figure 1:**
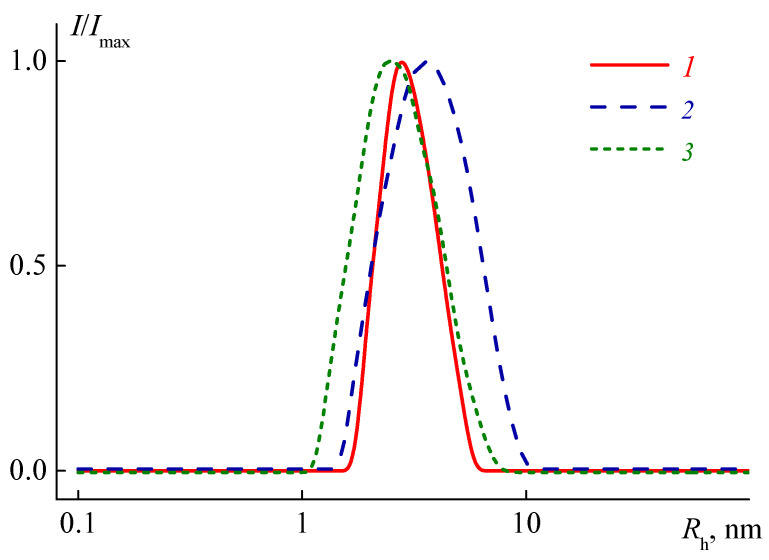
The dependencies of relative intensity *I*/*I*_max_ of scattered light on the hydrodynamic radii *R*_h_ of scattering species for 2-nitropropane solutions of C8A-(PiPrOx-b-PEtOx) at *c* = 0.040 g·cm^−3^ (1) C8A-(PEtOx-b-PiPrOx) at *c* =0.063 g·cm^−3^ (2) and C8A-P(EtOx-grad-iPrOx) at *c* = 0.069 g·cm^−3^ (3) *I*_max_ is the maximum value of light scattering intensity *I* at a given polymer concentration.

The distribution of the light scattering intensity *I* over the hydrodynamic radii *R*_h_ of the particles present in the solutions was unimodal (Figure 1). Within the studied concentration range, radii *R*_h_(*c*) depended on concentration *c*. Therefore, to determine the hydrodynamic radius *R*_h-D_ of macromolecules, the *R*_h_(*c*) values were extrapolated to zero concentration (Figure S1). The diffusion coefficients *D*_0_ of macromolecules were calculated according to the Stokes–Einstein equation using the obtained values of *R*_h-D_

*D*_0_ = *kT*_a_/(6πη_0_*R*_h-D_) (1)

where *k* is the Boltzmann constant and *T*_a_ is the absolute temperature.

For all studied polymer solutions, there was no light scattering asymmetry; therefore, the weight average molar mass *M*_w_ and the second virial coefficient *A*_2_ were found by the Debye method, taking measurements at a scattering angle 90°:

*cH*/*I*_90_ = 1/*M*_w_ + 2*A*_2_*c* (2)

where *I*_90_ is the light scattering intensity for an angle of 90° and *c* is the solution concentration. Optical constant *H* is calculated by the formula

**Figure 2:**
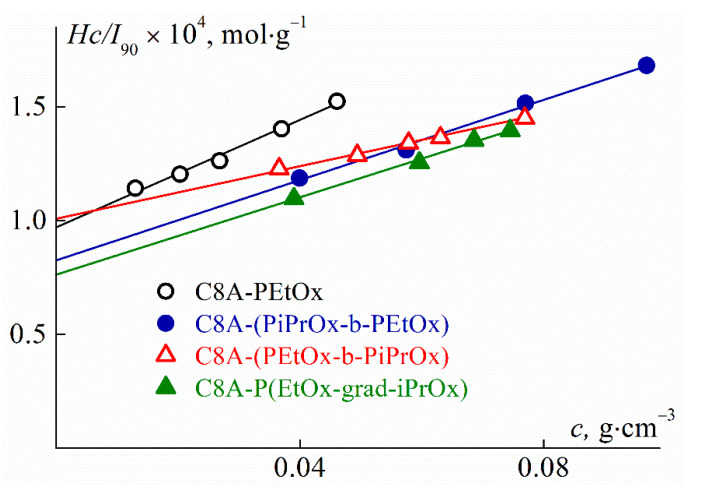
Concentration dependences of *Hc*/*I*_90_ for the CA8-PAlOx solutions in 2-nitropropane.

*H* = 4π^2^*n*_0_^2^(*dn*/*dc*)^2^/*N*_A_λ^4^ (3)

where *dn*/*dc* is the refractive index increment and *N*_A_ is the Avagadro number. Figure 2 shows the Debye dependencies for the studied star-shaped polymers. They are typical for dilute polymer solutions. The obtained values of *M*_w_ and *A*_2_ are listed in Table 1. Note that positive values of the second virial coefficient indicate a good thermodynamic quality of 2-nitropropane for the studied polymer stars. The refractive index increment *dn*/*dc* was measured on the RA-620 refractometer (KEM, Tokyo, Japan). The *dn*/*dc* values (Table 1) were determined from the slope of the concentration dependence of the difference *dn* = *n* − *n*_0_ in the refractive indices of solutions *n* and 2-nitropropane *n*_0_ (Figure S2).

Ostwald-type glass viscometers (Cannon Instrument Company Inc., State College, PA, USA) were used to measure intrinsic viscosity [η]. The solution temperature was regulated by a thermostat with a temperature control unit T-100 (Grant, Cambridge, UK). The solvent efflux time was 59.4 s. The concentration dependencies of the reduced viscosity η_sp_/*c* (Figure S3) were analyzed using the Huggins equation:

η_sp_/*c* = [η] + *k*′[η]^2^*c* (4)

where *k*′ is the Huggins constant. High *k*′ values, from 1.4 to 2.4, were obtained for the studied polymers. Note that increased values of the Huggins constant are often reported for not very high molecular mass samples of polymers with increased intramolecular density [41,42]. Using the obtained values of the intrinsic viscosity [η], the so-called viscosity hydrodynamic radius *R*_h-η_ of macromolecules were calculated by Einstein’s formula:

*R*_h-η_ = (3*M*[η]/(10πN_A_))^1/3^ (5)

### 2.9. Investigation of Self-Organization in Aqueous Solutions

The thermosensitive behavior of aqueous solutions of CA8-PAlOx was studied by light scattering and turbidimetry methods using the Photocor Complex setup described above. The experiments were carried out in a wide range of concentrations and temperatures. The temperature *T* was changed discretely with a step from 0.5 to 5 °C; the value of *T* was regulated with an accuracy of 0.1 °C. The measurement procedure is described in detail in [43]. After the given temperature was established, the dependencies of the light scattering intensity *I* and the optical transmittance I* on time *t* were obtained at a scattering angle of 90°. The hydrodynamic radii *R*_h_ of the scattering objects and their contribution *S*_i_ into total solution intensity *I* were determined when the values of *I* and I* became constant in time. These measurements were carried out at scattering angles from 45° to 135° to confirm the diffusion nature of the modes and obtain the extrapolated values of *R*_h_ and *S*_i_. The laser power changes from 5 to 30 mV and/or optical filters placing on the photodetector allowed one to attenuate the light scattering signal to 1.5 MHz and maintain the linearity of the device regarding *I.*

Before static and dynamic light scattering experiments, the solutions, 2-nitropropane and calibration liquid, toluene, were filtered through the Millipore syringe filter (Merck, Germany) with a pore diameter of 0.20 μm. The water solutions were filtered through hydrophilic PTFE Millipore (Merck, Germany) membrane filters with a pore diameter of 0.45 μm.

## 3. Results and Discussion

### 3.1. Synthetic Approach

**Figure 3:**
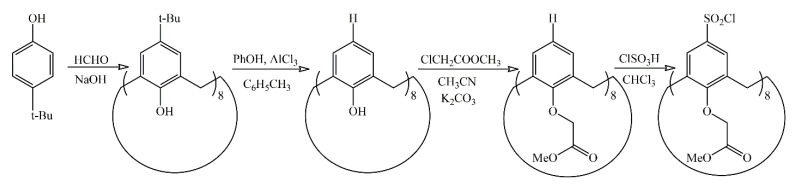
Synthesis of the octafunctional macrocyclic initiator.

Star-shaped poly(2-alkyl-2-oxazoline) block copolymers were synthesized using a “grafting from” approach. At first, the octafunctional macrocyclic initiator was prepared based on the tert-butyl-calix[8]arene. The lower rim of macrocycle was modified with ester groups to increase solubility, whereas the upper rim was functionalized with initiating sulfonyl chloride moieties. The reaction scheme is presented in Figure 3. Since aromatic sulfonyl chlorides were shown to be effective initiators of oxazoline polymerization [44], this approach was successfully applied in the present paper. The kinetic studies of 2-ethyl-2-oxazoline polymerization initiated by the obtained abovementioned calixarene initiator was reported [45] and it showed that the initiation reaction is rapid and chain growth proceeds via the “living chain” mechanism.

### 3.2. Polymer Synthesis

**Figure 4:**
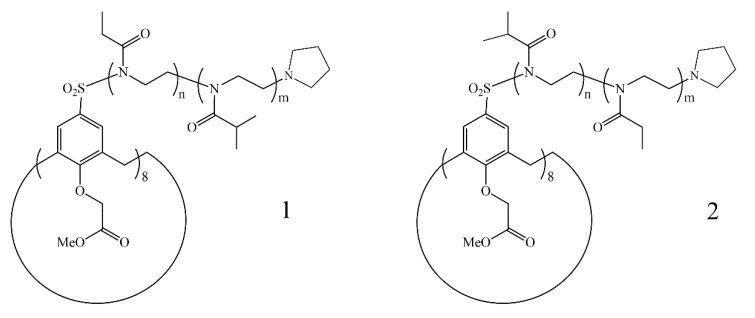
Structures of obtained star-shaped block-copolymers C8A-(PEtOx-b-PiPrOx) (**1**) and C8A-(PiPrOx-b-PEtOx) (**2**).

Monomers of 2-ethyl- and 2-isopropyl-2-oxazolines with the optimal hydrophobic–hydrophilic balance were chosen to obtain thermosensitive polymers. Sulfolane was chosen as the solvent keeping in mind the high rate of oxazoline polymerization in this solvent [46] and the enough solubility of the initiator in sulpholane. The post-polymerization technique was applied to obtain 8-arm star-shaped block-copolymers. After the complete consumption of the first type monomer, the polymerization was reinitiated by injection of the second type monomer. Two samples of block-copolymers were synthesized with a different order of blocks, namely, CA8-(PEtOx-b-PiPrOx) and CA8-(PiPrOx-b-PEtOx) (Figure 4). Monomers were taken in equivalent molar amounts to obtain polymeric blocks with equal lengths. It was shown, that amine-type terminating agents are the most preferred for 2-oxazoline polymerization because of rapid termination on the 5-position of the oxazoline ring [47]. Therefore pyrrolidine was used as the termination agent.

**Figure 5:**
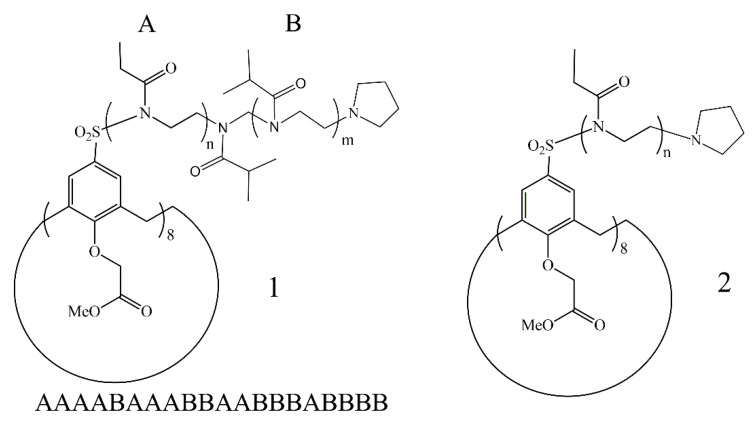
Structures of obtained star-shaped C8A-P(EtOx-grad-iPrOx) (**1**) and C8A-PEtOx (**2**).

In order to obtain the statistical copolymer, equivalent amounts of 2-ethyl-2-oxazoline and 2-isopropyl-2-oxazoline were mixed in the polymerization vial. The proposed method of synthesis consisted of the simultaneous copolymerization of both monomers. It was found that the relative reactivity of EtOx and iPrOx in simultaneous copolymerization are equal to 0.79 and 1.78, respectively [48]. It can be assumed that the structure of the statistical copolymer formed in the reaction mixture would be the gradient most probably. Figure 5 shows the structures of C8A-P(EtOx-grad-iPrOx) and star-shaped homopolymer C8A-PEtOx.

It is well known that the only parent calix[8]arene exists in the stable cone conformation due to the intramolecular H-bonding of hydroxyl groups at the lower rim while any kinds of chemical transformation of these moieties leads to conformationally labile structures that are clearly visible in the NMR spectra. It was found that methylene protons of the macrocycle did not exhibit the AB quartet that is typical for the core conformer and had no more complicated signals that are typical for paco or the other conformers [40]. Broad singlet at about 4 ppm verified the quick rotation in macrocycle. On the other hand functional moieties at the lower rim really define the complexation ability of the macrocycle that keeps in mind that the probable biomedical applications is the goal of our research.

### 3.3. Characterization of Polymers

**Figure 6:**
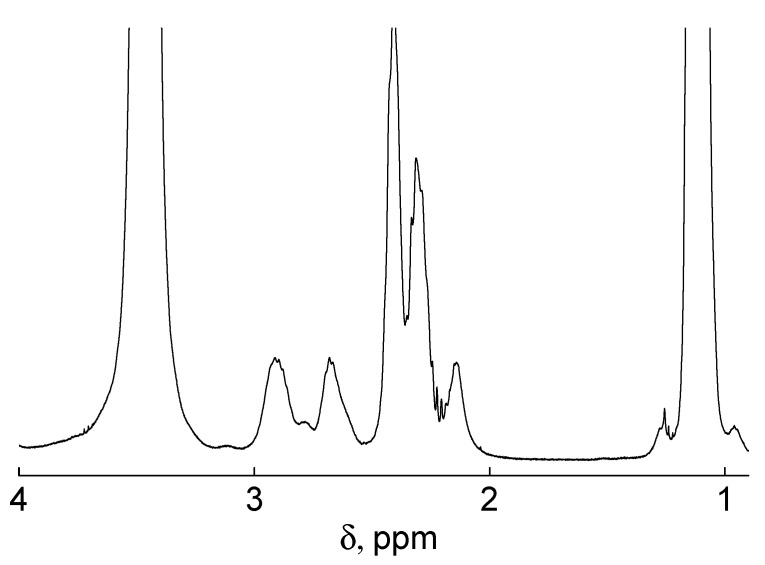
^1^H NMR spectrum of star-shaped C8A-(PEtOx-b-PiPrOx).

The spectra of all samples are completely similar and contain signals of both ethyl (2.50–2.20 ppm) and isopropyl (3.05–2.55 ppm) groups, which confirms the presence of both monomers in the polymer chains (Figure 6). Additionally, minor signals of the calix[8]arene core, attributed to the bridged methylene groups (4.46 ppm) were detected and ester methylene groups at the lower rim (4.23–3.97 ppm). According to the NMR data the integral intensities of proton signals at about 2.3 ppm (CH_2_CH_3_ in ethyloxazoline) and doublet at about 2.5 and 2.8 (CH(CH_3_)_2_ in isopropyloxazoline) are 2:1. Based on the integral intensities of proton signals, it was determined that the ratio of the monomer in the copolymer was at about 1:1 for all samples. Therefore, both blocks have a near equal degree of polymerization. The same ratio of components was calculated for the gradient copolymer sample. This conclusion is confirmed by the fact that the values of the refractive index increment dn/dc coincide within the experimental error for stars with copolymer arms (Table 1) but lower than dn/dc for C8A-PEtOx. This behavior was observed for PAlOx copolymers [25,39,49] and is explained by an increase in the refractive index on the passage from polymers with ethyl groups to samples containing isopropyl ones.

The presence of calix[8]arene cores in the synthesized copolymers was also confirmed by UV–visible spectroscopy (Figure S4). The typical absorption bands at about 250–290 cm^−1^ confirm the presence of the macrocycles core in the polymer structure.

The number of arms in a star-shaped polymer was determined by the selective destruction of the macromolecule without degradation of the poly-2-alkyl-2-oxazoline original length. For this purpose, it was applied the original procedure involving acid hydrolysis of sulfonamide groups to polyethylenimine followed by acylation with propionic anhydride and GPC analysis of the obtained oligomers. Using the MM of star-shaped polymers and their arms, the arm number fa was calculated. The fa values are at about 8, i.e., all polymers have an eight-arm structure.

The arms of the synthesized stars are short (Table 2). Their length *L*_a_ was calculated by the ratio:

*L*_a_ = *N*_a_λ_a_ = λ_a_(*M*_w_ − *M*_C8A_)/*f*_a_*M*_0-a_ (6)

where *f*_a_ = 8 is the arm number, *N*_a_ is the polymerization degree of arms, λ_a_ = 0.378 nm is the length of the monomer unit of poly-2-alkyl-2-oxazoline [50], and *M*_C8A_ = 1928 g·mol^–1^ is the molar mass of CA8. In the case of copolymers, the average molar mass (MM) of the arm monomer units was equal to 106 g·mol^−1^, i.e., the average value of MM of 2-ethyl-2-oxazoline (99 g·mol^–1^) and 2-isopropyl-2-oxazoline (113 g·mol^−1^). Poly-2-ethyl- and poly-2-isopropyl-2-oxazoline were comb-shaped with short side chains containing three valence bonds to the point of the most distant from the backbone chain and differing by one –CH_3_ group. Systematic studies of various classes of comb-shaped polymers [51] showed that, with those insignificant structural variations, the conformational characteristics of polymers almost do not change. Therefore, it can be assumed that the Kuhn segment lengths for blocks of poly-2-ethyl- and poly-2-isopropyl-2-oxazoline are equal to *A* = (1.4 − 1.8) nm [50,52]. Accordingly, using the average value *A* = 1.6 nm, we can estimate the number N* of Kuhn segments in the arms of the studied polymer stars. From Table 2 it is seen that N* did not exceed 3.

### 3.4. Hydrodynamic Characteristics and Conformation of Star-Shaped CA8-PAlOx-UR

Gel permeation chromatography (Figure S5) shows that all samples are characterized by a monomodal molar mass distribution. This behavior is in qualitative agreement with the dynamic light scattering data obtained in molecularly dispersed solutions in a wide range of polymer concentrations. The polydispersity indexes *Đ* = *M*_w_/*M*_n_ of studied star samples are shown in Table 1.

It should also be noted that the molar masses of the synthesized star-shaped polymers differed insignificantly, the maximum difference was about 30%. Therefore, in further analysis and comparison of the results obtained, the influence of MM on the polymer characteristics could be neglected.

The hydrodynamic radii of CA8-PAlOx molecules are less than the arm lengths *L*_a_ (Table 1 and Table 2). This indicates that the macromolecules were compact and the arms, despite their small length, were relatively strongly folded. The compact structure of CA8-PAlOx molecules was also confirmed by the low values of intrinsic viscosity [η]. Table 2 shows the values of the viscosity contraction factor.

*g*′ = [η]_star_/[η]_lin_ (7)

where [η]_star_ and [η]_lin_ are the characteristic viscosities of star-shaped and linear polymers of the same MM. As the [η]_lin_ values, we used the average values [η] for linear poly-2-ethyl-2-oxazoline, calculated from the Mark–Kuhn–Houwink–Sakurada equations obtained in thermodynamically good solvents [50,53]. Moreover, the conformation and hydrodynamic properties of linear PEtOx and PiPrOx could be assumed identical. This conclusion is supported by the results of studies of linear and star-shaped poly(2-ethyl-2-oxazine) [54]. It was shown that a change by one –CH_2_– group of the monomer unit of pseudo-polypeptoids does not lead to a change in the conformational characteristics of the polymer.

The behavior of contraction factor *g* = (*R*_g_)_star_^2^/(*R*_g_)_lin_^2^, determined from the ratio of the squared gyration radii of star-shaped (*R*_g_)_star_ and linear (*R*_g_)_lin_ polymers, has been theoretically analyzed in detail. Zimm and Stockmayer [55] showed polymer stars with long monodisperse arms:

*g* = (3*f*_a_ − 2)/*f*_a_^2^ (8)

Therefore, *g* = 0.34 for a star with eight arms. In the case of polydisperse arms [56,57]:

*g* = 3*f*_a_/(*f*_a_ + 1)^2^, (9)

where *g* = 0.30 for eight-arm stars. Daoud–Cotton theory [58] describes *g* for multiarm star-shaped polymers with short arms as

*g* = *f*_a_^−4/5^, (10)

where the contraction factor is *g* = 0.19 at *f*_a_ = 8. For the studied CA8-PAlOx, the value of g can be calculated using the empirical equation [59]

*g*′ = (1.104 − 0.104*g*^7^)*g*^0.906^ (11)

The g values for CA8-PAlOx with copolymer arms lie between the theoretical values of the contraction factor for star-shaped macromolecules with short and long arms (Table 2). This behavior is in agreement with the findings of the study of six-arm polypeptoids [49], which can be considered as long-arm molecules if the arms contain more than six Kuhn segments. A higher value of *g* was obtained for CA8-PEtOx, the reason for which remains unclear.

The low hydrodynamic invariant *A*_0_ [51,60,61]:

*A*_0_ = (η_0_*D*_0_(*M*[η]/100)^1/3^/*T*_a_ (12)

where it justifies the compact structure of the molecules of studied CA8-PAlOx. The obtained values of *A*_0_ (Table 1) were less than 3.2 × 10^−10^ erg·K^−1^·mol^−1/3^, predicted theoretically for flexible chain polymers [51,60]. In particular, for linear poly-2-ethyl-2-oxazoline the average value of hydrodynamic invariant is 3.1 × 10^−10^ erg·K^−1^·mol^−1/3^ [58]. On the other hand, the *A*_0_ values for the star-shaped CA8-PAlOx are noticeably larger than the hydrodynamic invariant for dendrimers and hyperbranched polymers [62,63,64], which are polymers with high intramolecular density. Similar values of *A*_0_ were obtained previously for star-shaped polypeptoids with short arms [49]. Accordingly, analysis of the hydrodynamic invariant makes it possible to conclude that polymer stars occupy an intermediate position between linear flexible chain polymers and dendritic systems in terms of the intramolecular density.

### 3.5. Self-Organization of C8A-PAlOx-UR Molecules in Aqueous Solutions

**Figure 7:**
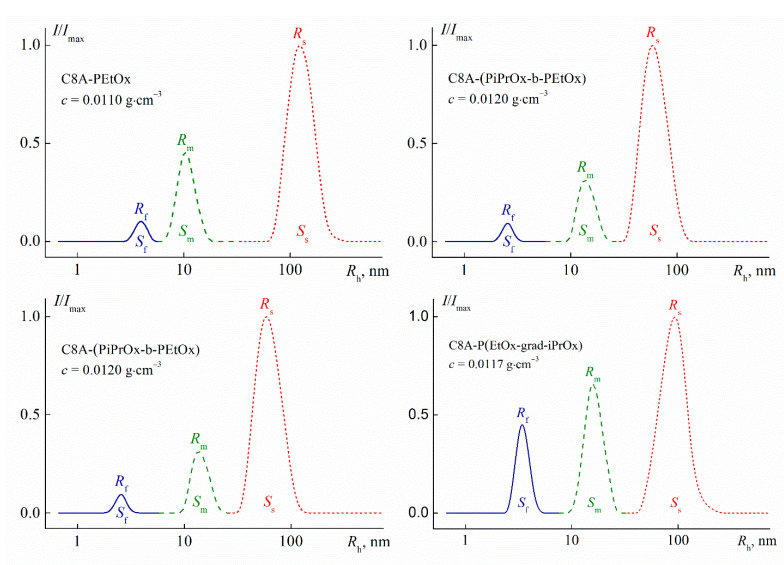
The dependences of relative intensity *I*/*I*_max_ of scattered light on the hydrodynamic radius *R*_h_ of scattering species for aqueous solutions of C8A-PAlOx at 21 °C. *I*_max_ is the maximum value of light scattering intensity for the given solution concentration.

**Figure 8:**
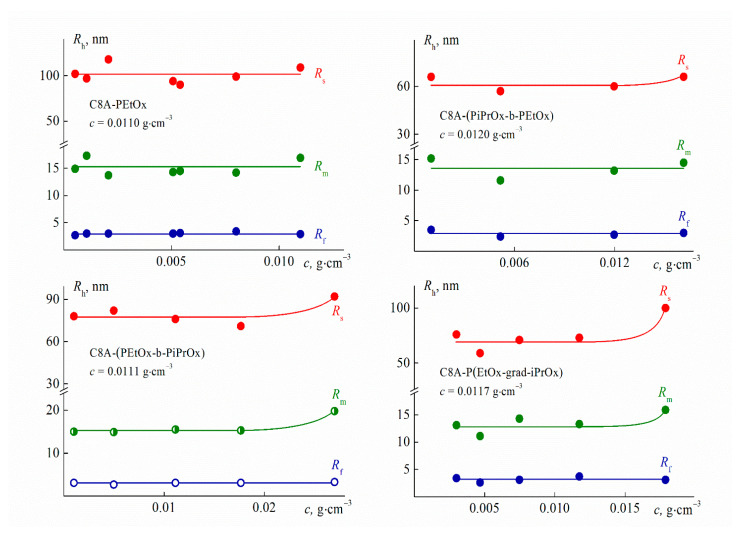
Concentration dependences of hydrodynamic radii *R_h_* of scattering species for aqueous solutions of C8A-PAlOx at 21 °C.

For all studied solutions at low temperatures, three modes with hydrodynamic radii *R*_f_ (fast mode), *R*_m_ (middle mode), and *R*_s_ (slow mode) were observed (Figure 7). *R*_f_ values did not vary with concentration (Figure 8) and the concentration-average *R*_f_ value coincided with the size *R*_h-D_ of macromolecules for each polymer and so the particles responsible for the fast mode are single macromolecules. The middle mode and slow mode reflect the diffusion of aggregates similar to those formed in solutions of thermoresponsive polymers [65,66,67,68,69,70,71,72,73,74,75,76], including star-shaped PAlOx [25,49]. The reason for the formation of aggregates is the interaction of hydrophobic cores.

The *R*_m_ and *R*_s_ values display an independence of concentrations up to *c* ≈ 0.015 g·cm^–3^, above which their slight growth is observed (Figure 8). As known, a change in the hydrodynamic radii can be caused both by a change in size of scattering species and by the concentration dependence of the diffusion coefficient *D*. The dependence *D*(*c*) is determined by the values of the second virial coefficient, molar mass, concentration coefficient of sedimentation, and specific partial volume. Probably, these factors make up for each other at low concentrations, when the aggregate dimensions increase and the coefficient *D* reduces. Consequently, *R*_m_ and *R*_s_ were constant at *c* < 0.015 g·cm^−3^.

One can see in Figure 7, the largest contribution *S_s_* to light scattering is made by large aggregates with a radius *R_s_*, while the contribution S_f_ of macromolecules is minimal. Nevertheless, the latter prevails in the solution. Indeed, the contribution *I_i_* = *S_i_I* of *i*th set of particles to the total light scattering intensity *I* is described by the relation *I_i_* ~ *c_i_R_i_^x^*, where *c_i_* and *R_i_* are the weight concentration and radius of the *i*th particles, respectively [77,78]. The value of the exponent *x* depends on the shape of the scattering particles. The fraction of each type of particles in solutions of the studied polymers can be roughly estimated using the models of a hard sphere (molecules and aggregates with a radius *R_m_*, *x* = 3) and a coil (large aggregates, *x* = 2). This approach is supported by the results of the conformational analysis of multiarm stars with short arms [58,79,80] and the studying micelle-like and large aggregates [71,72,73,74,75,81]. An estimation shows that the relative fraction *c_f_*/*c* of molecules in solutions of the C8A-(PiPrOx-b-PEtOx) and C8A-(PEtOx-b-PiPrOx) stars with block copolymer arms was about 80% (*c_f_* is the concentration of macromolecules in solution). The *c_f_*/*c* ratio increased up to 87% for the homopolymer C8A-PEtOx and 98% for the gradient C8A-P(EtOx-grad-iPrOx). Note that, the weight fraction *c_s_*/*c* of large aggregates did not exceed 10% for solutions of block copolymers and *c_s_*/*c* < 0.1% for the two other stars (*c_s_* is the concentration of large aggregates with a hydrodynamic radius *R_s_*).

**Figure 9:**
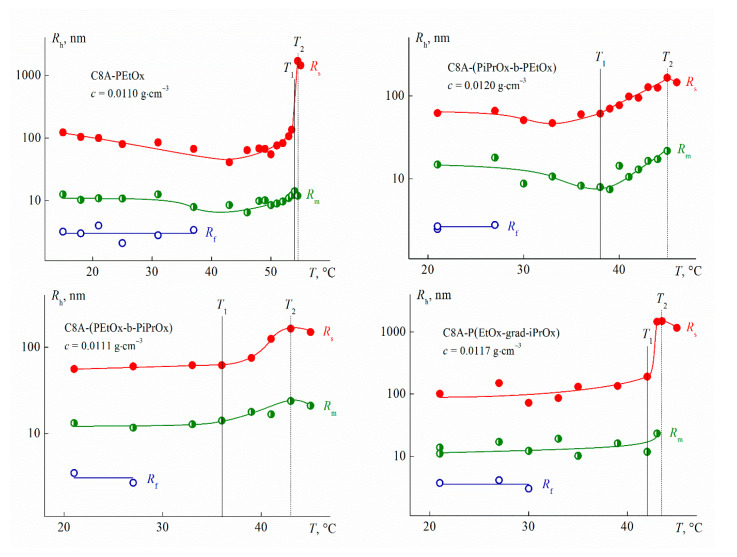
Temperature dependences of hydrodynamic radii *R_h_* of scattering particles for aqueous solutions of C8A-PAlOx.

Thus, the arm structure did not significantly affect the set of scattering objects and the hydrodynamic radii of aggregates, which is in opposition to results for star-shaped PAOx with copolymer arms grafted to a lower rim of the calix[8]arene [25,81]. This is probably due to both the more pleated loop conformation of C8A, functionalized along the upper rim [39], and the short arm length of the studied star samples. These factors lead to a decrease in the shielding of the core surface by arms, which promotes the aggregation. Probably, this is a reason why macromolecules disappear, or rather, have not been observed by the method of dynamic light scattering upon solution heating at relatively low temperatures, *T* ≤ 37 °C, far from the phase separation interval (Figure 9).

**Figure 10:**
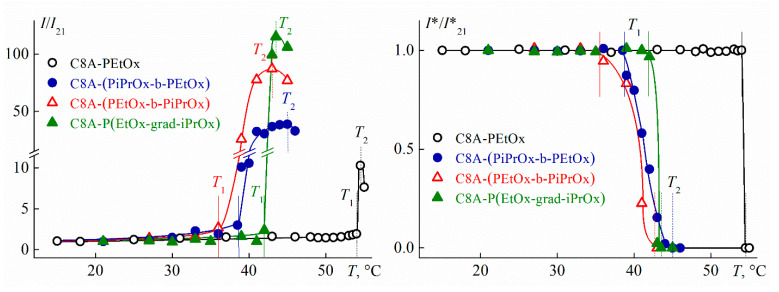
Dependencies of relative optical transmission I*/I*_21_ and relative light scattering intensity *I*/*I*_21_ on temperature *T* for investigated polymer stars. I*_21_ and *I*_21_ are the optical transmission and light scattering intensity at 21 °C, respectively. The concentration of the solutions is the same as in Figure 7, Figure 8 and Figure 9.

The temperatures of the onset *T*_1_ and the finishing *T*_2_ of the phase separation were determined by turbidimetry (Figure 10). Optical transmission I* did not depend on the temperature up to *T*_1_. On the contrary, the scattered light intensity *I* varies over the whole temperature range (Figure 10). At low temperatures, solution heating is accompanied by a slow *I* growth. The rate of change in intensity *I* dramatically increases at temperature *T*_1_. Light scattering intensity reached a maximum value at *T*_2_ and decreased slightly upon further heating for most of the studied solutions. 

The *I*(*T*) dependence was caused by changes in the size of the aggregates and their fraction in the solution with temperature. As mentioned above, macromolecules cease to be detected well below *T*_1_, which indicates the aggregation of macromolecules at *T* < *T*_1_, probably, caused by the dehydration of iPrOx units, which begins at relatively low temperatures [68]. As can be seen in Figure 9, for stars C8A-(PEtOx-b-PiPrOx) and C8A-P(EtOx-grad-iPrOx) on the periphery of which the more hydrophobic iPrOx units prevail, the hydrodynamic radii *R_s_* of large aggregates increased with increasing temperature. At low temperatures, this change was very weak and greatly accelerated at temperature *T*_1_ and the *R_s_* values reached hundreds and even thousands of nanometers at *T*_2_. In this case, the contribution *S_s_* of large aggregates to the integral light scattering increased owing to a lowering in the contribution *S_m_* of aggregates with a radius *R_m_*. A similar behavior was observed earlier for four- and eight-pointed stars with PiPrOx arms grafting to the lower rim of calix[n]arene [79]. For solutions C8A-PEtOx and C8A-(PiPrOx-b-PEtOx) a decrease in *R_s_* is observed at moderate temperatures (Figure 9). This change did not exceed 30% in comparisons with *R_s_* value at 21 °C. A decrease in *R_s_*, i.e., compaction of large aggregates, indicates the formation of inter- and intramolecular hydrogen bonds between dehydrated iPrOx units in one aggregate scale. Note that the *R_s_* value decreased much more, sometimes more than ten times, for stars with a copolymer PAlOx arm grafted to the lower rim of C8A and for PiPrOx stars with carbosilane dendrimer cores [25,26,82]. The platter conformation of C8A and shorter arms in the studied polymer stars facilitated contacts between hydrophobic cores of different molecules, which should lead to aggregation. Indeed, the contributions *S_s_* of large aggregates to light scattering increased with temperature for C8A-PEtOx and C8A-(PiPrOx-b-PEtOx). For example, for a C8A-PEtOx solution at a concentration of *c* = 0.0110 g∙cm^−3^, the *S_s_* magnitude increased from 0.45 at 21 °C to 0.66 at a temperature of minimum value of *R_s_*, and *S_s_* varies from 0.81 to 0.94 for C8A-(PiPrOx-b-PEtOx) at *c* = 0.0120 g∙cm^−3^. Concerning smaller aggregates in solutions of the stars under discussion, the *R_m_* value changes with temperature in the same way as the radius of *R_s_*, but this change is within the experimental error (Figure 9). The dimensions of large aggregates reached a maximum value within the interval of phase separation and then decreased, reflecting the compaction of these particles. However, it should be taken into account that under these conditions, light scattering was multiple and a quantitative analysis of the results was impossible.

**Figure 11:**
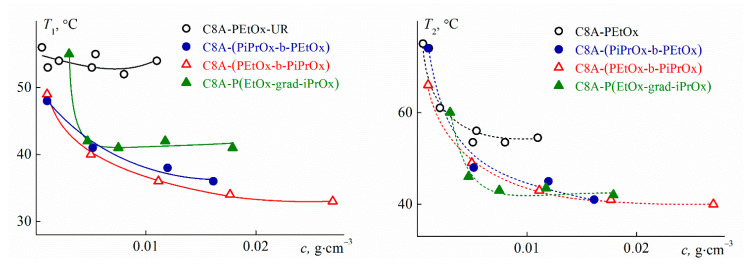
Phase transition temperatures vs. polymer concentration for investigated polymer solutions.

One phase transition was observed for all studied star-shaped polymers at all concentrations. Figure 11 shows the phase separation temperatures versus concentration. As expected, *T*_1_ and *T*_2_ depended on the arm structure. The highest values of *T*_1_ and *T*_2_ were obtained for C8A-PEtOx, which were 15–20 °C lower than *T*_1_ and *T*_2_ for the eight-arm star with the PEtOx arm grafted to the lower rim of C8A [83] at the same concentrations.

The introduction of a more hydrophobic block of PiPrOx into the star macromolecules led to a decrease in the *T*_1_ and *T*_2_ values for solutions of C8A-(PEtOx-b-PiPrOx), C8A-(PiPrOx-b-PEtOx) and C8A-P(EtOx-grad-iPrOx) as compared to C8A-PEtOx solutions. The temperatures *T*_1_ of the phase separation onset for stars with copolymer arms differed and the temperatures *T*_2_ of its finishing product almost coincided. Similar results were obtained for the copolymer PAlOx stars with the C8A core functionalized along the lower rim [25], for which, however, the values of *T*_1_ and *T*_2_ were significantly higher. The pattern of the concentration dependencies of the phase separation temperatures for the star-shaped polymers under consideration differed markedly. In particular, an LCST of about 41 °C can be determined reliably for the star C8A-P(EtOx-grad-iPrOx). The flattening of the *T*_1_(*c*) dependence for C8A-(PEtOx-b-PiPrOx) in the region of high concentrations allows suggesting that LCST was close to 32–33 °C, that is almost 10 °C lower than for C8A-P(EtOx-grad-iPrOx). The *T*_1_ values for the block copolymer star with a more hydrophilic outer block changed quite strongly within the whole concentration range and it was not possible to estimate the LCST for C8A-(PiPrOx-b-PEtOx), but it was unlikely that it differed greatly from 30 °C.

At the same concentrations, the phase separation temperatures for the studied copolymer stars are several degrees higher than *T*_1_ and *T*_2_ for solutions of a star with a C8A core functionalized along the lower rim by PiPrOx chains [79]. It is expected given that the dehydration temperature of PEtOx is higher than for PiPrOx [68]. On the other hand, despite the presence of hydrophilic EtOx units in arms, the LCSTs for solutions of the studied stars with copolymer arms are near to the LCST of linear PiPrOx [65,84]. The latter can be explained by both the distinction in molecule architecture and hydrophobic C8A core and different MMs of the compared polymers.

All of the results discussed were obtained, when the characteristics of the solution reached constant values in time after a jump-like temperature change. The analysis of the processes of establishing the “equilibrium” state of the system, in particular, determining their duration depending on the chemical structure of the polymer and external conditions are an important task since the rate of change of the characteristics of the stimulus-sensitive polymer solution determines the application field of the material based on it. Nevertheless, the number of works devoted to solving this problem remains small [43,68,85,86,87,88,89,90] and many questions remain open.

Figure S6 shows the dependencies of the scattered light intensity I and optical transmission I* on time t after the discrete temperature change. The moment when the temperature reaches a given value is taken as *t* = 0. The intensity *I* and the transmission I* reach a constant value in time *t*_eq_.

**Figure 12:**
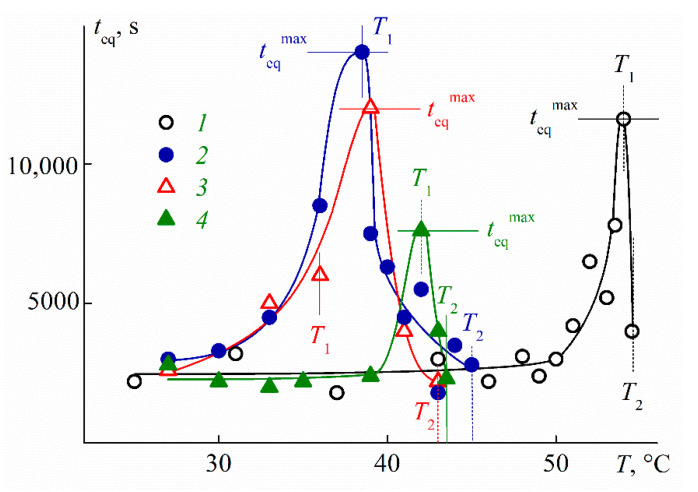
Dependences of time *t*_eq_ on temperature *T* for aqueous solutions of C8A-PEtOx (1) at concentration *c* = 0.0110 g∙cm^−3^, C8A-(PiPrOx-b-PEtOx) (2) at *c* = 0.0120 g∙cm^−3^, C8A-(PEtOx-b-PiPrOx) (3) at *c* = 0.0111 g∙cm^−3^, and C8A-P(EtOx-grad-iPrOx) (4) at *c* = 0.0117 g∙cm^−3^.

The duration of the processes of establishing the “equilibrium” state was long for the studied star-shaped C8A-PAlOx and strongly depended on the temperature at each concentration (Figure 12). At low temperatures, the *t*_eq_ values ranged from 1200 to 3000 s, which is higher than the usual ones for linear stimulus-sensitive polymers [68,85,86]. Time *t*_eq_ sharply increased upon heating and reached the maximum *t*_eq_^max^ value near the phase separation. At *T* > *T*_1_, t_eq_ fell and was about 1000 s at *T* → *T*_2_. A similar *t*_eq_(*T*) dependence was observed earlier for PAlOx of a complex architecture [43,90,91,92,93]. 

No systematic variation of *t*_eq_^max^ with concentration was found for the studied stars, and the *t*_eq_^max^ values were in the range from 8000 to 15,000 s. Similar times for the reaching “equilibrium” values of solution characteristics were obtained for star-shaped PAlOx with copolymer arms grafted to the lower rim of the calix[8]arene [25,81]. In the case of star-shaped PiPrOx, the time *t*_eq_^max^ strongly depends on the core configuration; the *t*_eq_^max^ value did not exceed 20,000 s when arms grafted to the upper rim of C8A and reached 40,000 s when C8A core was functionalized along the lower rim [43]. Thus, the rate of self-organization processes in solutions of PAlOx stars depends both on the way of functionalization of the calix[n]arene core, which changes its configuration, and on the structure of PAlOx arms because the presence of EtOx units led to a decrease in values of *t*_eq_^max^ as compared to star-shaped PiPrOx homopolymers.

## 4. Conclusions

Star-shaped poly-2-alkyl-2-oxazolines with arms of block and gradient copolymers of 2-ethyl- and 2-isopropyl-2-oxazolines grafted to the upper rim of calix[8]arene were synthesized by the “grafting from” method. The ratio of 2-ethyl- and 2-isopropyl-2-oxazoline units was 1:1, which was confirmed by NMR spectroscopy and refractometry. The arms of the synthesized stars were relatively short, their length did not exceed 5 nm. The arm conformation was folded in an organic solvent, and the star-shaped C8A-PAlOx macromolecules were characterized by compact dimensions and heightened intramolecular density. The influence of the arm structure on the conformation of C8A-PAlOx molecules was not observed.

At low temperatures, the aqueous solutions of the studied C8A-PAlOx-UR were not molecular dispersed; there were aggregates formed due to the interaction of hydrophobic calix[8]arene cores. Nevertheless, single macromolecules prevailed, its relative fraction always exceeded 80%. No systematic changes in the set of scattering objects and their dimensions were found for studied stars.

Below the phase separation temperature, the heating of aqueous solutions of C8A-PAlOx with prevailing iPrOx units in the outer layer caused the aggregation of macromolecules because of an increase in the degree of dehydration of iPrOx units with temperature. For stars with an outer PEtOx layer, there was a rather wide temperature interval, in which compaction prevailed.

One phase transition was detected for all studied polymer stars. The temperatures of its onset *T*_1_ and finish *T*_2_ decreased with a growth of the content of more hydrophobic iPrOx units. The highest temperatures were obtained for the homopolymer C8A-PEtOx, while they decreased by 10–20 °C for stars with copolymer arms. The distinction between the phase separation temperatures for copolymer stars and a homopolymer with PiPrOx arms was moderate. Consequently, iPrOx dehydration determines the *T*_1_ and *T*_2_ values. The arm structure did not affect the value of *T*_2_ but led to a change in the concentration dependence of *T*_1_. Thus the highest LCST could be expected for a star with gradient copolymer arms, while LCST for C8A-(PEtOx-b-PiPrOx)-UR and C8A-(PiPrOx-b-PEtOx)-UR were 10 °C lower.

The molar masses of synthesized samples were similar and the effect of MM on the solution behavior was not detected. The way of arms grafting to the core, at the lower or upper rim of C8A, significantly affected the behavior of star-shaped PAlOx in aqueous solutions. First of all, it is manifested in a decrease of the phase separation temperatures for stars with C8A, functionalized along the upper rim. The reason for the observed phenomenon is the different core configuration. When functionalization is along the lower rim, the arms tended to shield the hydrophobic C8A, enveloped it, and slightly constricted the upper rim of C8A. The arms grafted to the upper rim, on the contrary, increased its radius due to steric interactions. Thus, the shape of the C8A with arms at the lower rim resembled a basket, while the C8A with arms at the upper rim looks like a plate. A change in the core configuration varied its accessibility to the solvent and, consequently, the phase separation temperature.

## Figures and Tables

**Table 1:** Molar mass and hydrodynamic characteristics of star-shaped C8A-PAlOx.

Sample	*M_w_*, g·mol^−^^1^	*Đ*	*R*_h-D_, nm	[η], cm^3^·g^−^^1^	*R*_h-[η]_, nm	*dn/dc,* cm^3^·g^−^^1^	*A*_2_·10^4^, cm^3^·mol·g^−2^
C8A-PEtOx	10,300	1.38	2.6	8.2	2.4	0.1246	5.9
C8A-(PiPrOx-b-PEtOx)	12,100	1.21	2.4	5.9	2.2	0.1166	4.4
C8A-(PEtOx-b-PiPrOx)	10,000	1.35	2.0	4.9	2.0	0.1156	2.8
C8A-P(EtOx-grad-iPrOx)	13,200	1.41	2.9	7.7	2.5	0.1160	4.3

**Table 2:** Structure characteristics and contraction factors for star-shaped CA8-PAlOx.

Sample	*M_w_*, g·mol^−^^1^	*M_w_* (arm), g·mol^−^^1^	*f_a_*	*L*_a_, nm	N*	g′	*g*	*A*_0_ × 10^10^, erg·K^−1^mol^−1/3^
C8A-PEtOx	10,300	1100	8.0	4.0	2.4	0.45	0.37	2.7
C8A-(PiPrOx-b-PEtOx)	12,100	1300	8.0	4.5	2.8	0.29	0.23	2.7
C8A-(PEtOx-b-PiPrOx)	10,000	1050	8.1	3.6	2.3	0.27	0.21	2.9
C8A-P(EtOx-grad-iPrOx)	13,200	1400	7.6	4.7	2.9	0.34	0.27	2.7

## Data Availability

Not applicable.

## Supplementary Materials

The following are available online at https://www.mdpi.com/article/10.3390/polym13152507/s1, Figure S1: Plots of *R*_h-D_(*c*) vs. concentration *c* for the studied polymer stars in 2-nitropropane. Figure S2: Concentration dependencies of *dn* for the C8A-PaOx solutions 2-nitropropane. Figure S3: Reduced viscosity η_sp_/*c* vs. *c* for the studied polymer stars in 2-nitropropane. Figure S4: UV–visible spectrum of the star-shaped C8A-(PEtOx-b-PiPrOx)-UR solution in ethanol. Polymer concentration is 0.005 g·cm^−3^. Figure S5: GPC traces of star copolymers CA8-PAlOx. Figure S6: Time dependencies of light scattering intensity *I*/*I*_0_ and the transmission *I**/*I*_0_* for solutions C8A-PAlOx. *I*_0_ and *I**_0_ are values of light scattering intensity and the optical transmission at *t* = 0.
