# Supplementary material for: Features of Solution Behavior of Polymer Stars with Arms of Poly-2-alkyl-2-oxazolines Copolymers Grafted to the Upper Rim of Calix[8]arene
Source: Polymers (Basel). 2021 Jul 29;13(15):2507. doi: 10.3390/polym13152507 (PMC8348004; doi:10.3390/polym13152507)
Supplement: Supplementary file 1 [file polymers-13-02507-s001.zip › polymers-1298718-supplementary.pdf]

Supplementary Materials

Features of solution behavior of polymer stars with arms of poly-2-alkyl-2-oxazoline copolymers grafted to the upper rim of calix[8]arene

Tatyana Kirila [1,]*, Alina Amirova [1], Alexey Blokhin [1], Andrey Tenkovtsev [1], Alexander Filippov [1]

[1]Institute of Macromolecular Compounds of the Russian Academy of Sciences, Bolshoy pr., 31, Saint Petersburg, 199004 Russia

*Corresponding author, E-mail: tatyana_pyx@mail.ru

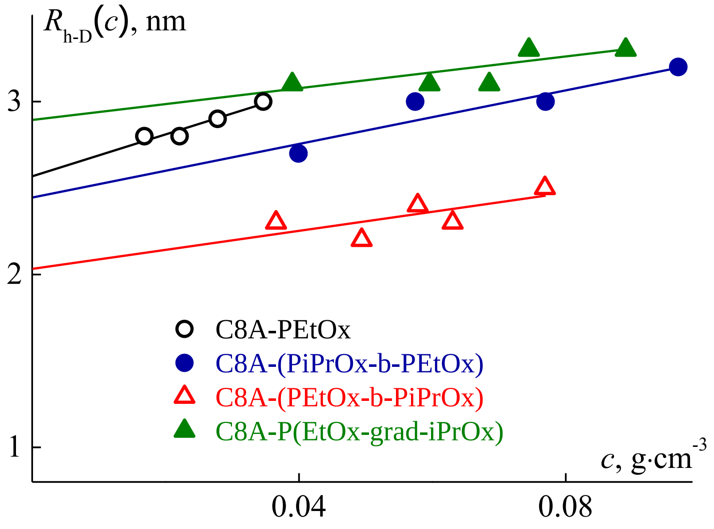

Figure S1. Plots of $R_{h\text{-}D}(c)$ vs. concentration c for the studied polymer stars in 2-nitropropane.

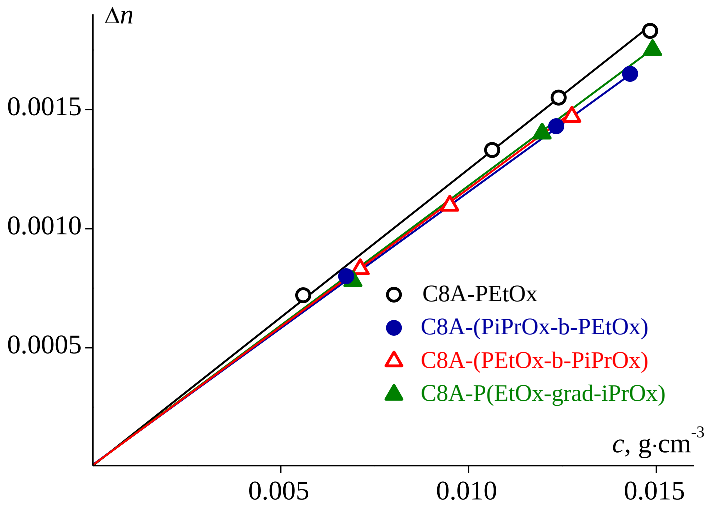

Figure S2. Concentration dependencies of dn for the C8A-PaOx solutions 2-nitropropane.

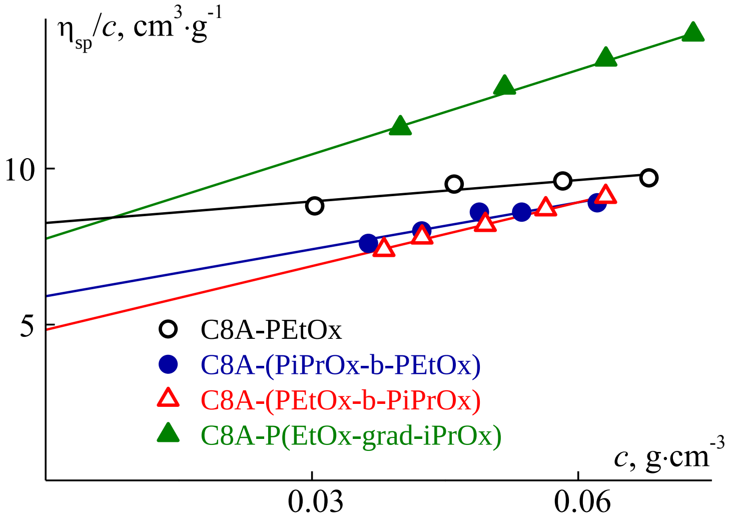

Figure S3. Reduced viscosity η_{sp}/c vs. c for the studied polymer stars in 2-nitropropane.

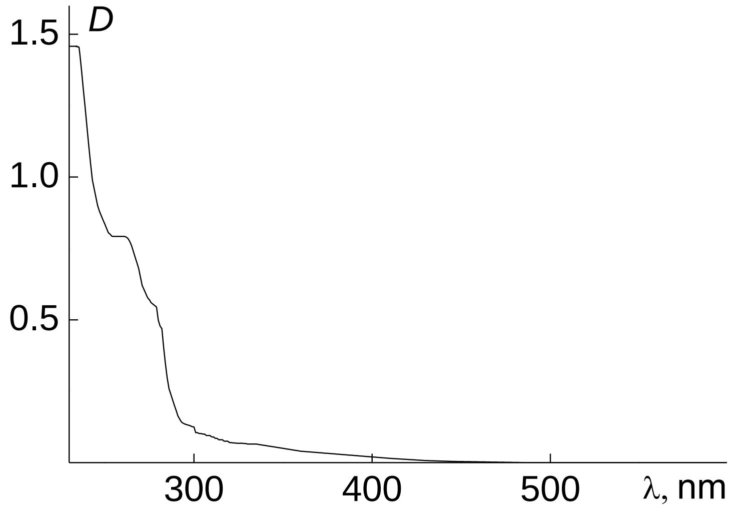

Figure S4. UV-visible spectrum of the star-shaped C8A-(PEtOx-b-PiPrOx)-UR solution in ethanol. Polymer concentration is 0.005 g·cm^{-3}.

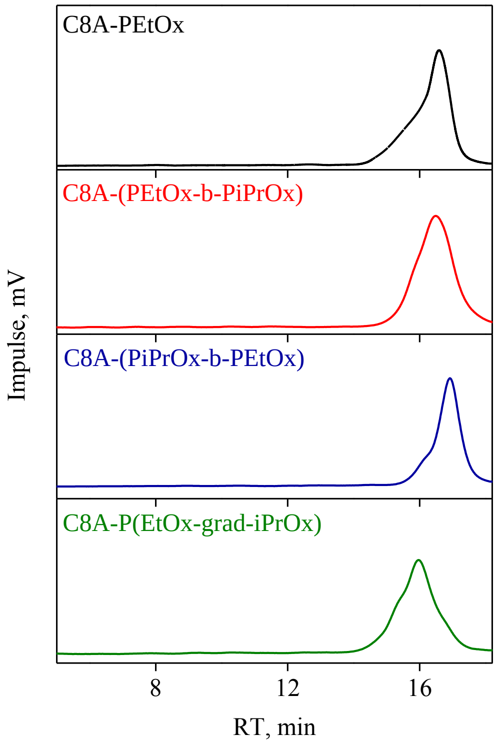

Figure S5. GPC traces of star copolymers C8A-PAlOx.

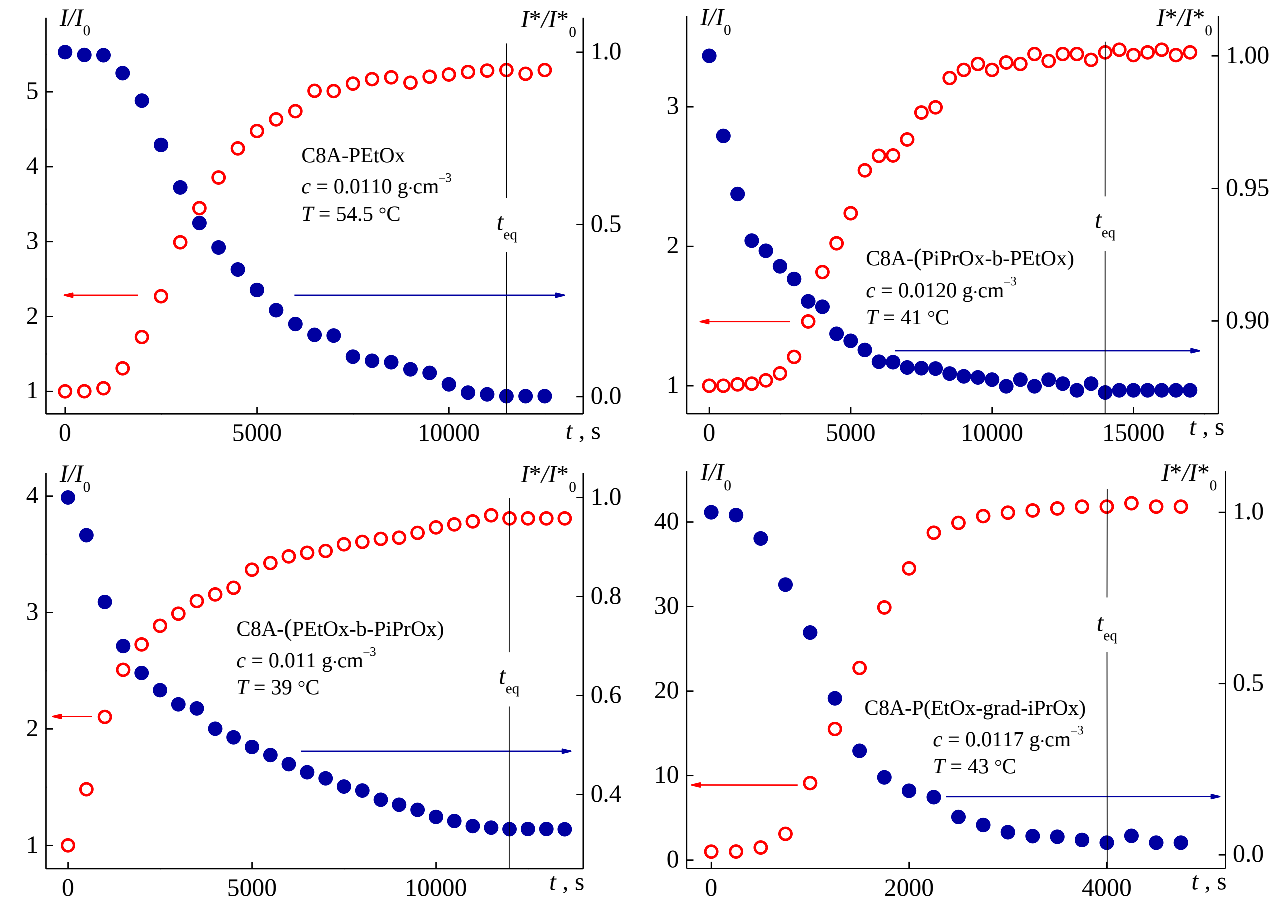

Figure S6. Time dependencies of light scattering intensity I/I_0 and the transmission I^*/I_0^* for solutions C8A-PAlOx. I_0 и I^*_0 are values of light scattering intensity and the optical transmission at $t = 0$.
